# Effects of whole-body vibration therapy on pain, functionality, postural stability, and proprioception in patients with subacute and chronic non-specific low back pain: a systematic review

**DOI:** 10.1007/s10354-023-01026-4

**Published:** 2023-11-24

**Authors:** Franziska Remer, Mohammad Keilani, Philipp Kull, Richard Crevenna

**Affiliations:** Department of Physical Medicine, Rehabilitation and Occupational Medicine, Medical University of Vienna, Waehringer Guertel 18–20, 1090 Vienna, Austria

**Keywords:** Whole-body vibration treatment, Back pain, Pain reduction, Physical function, Sensimotor function, Ganzkörper-Vibrationstherapie, Rückenschmerzen, Schmerzreduktion, Funktionalität, Sensomotorik

## Abstract

**Introduction:**

Non-specific low back pain (NLBP) is a common and clinically significant condition with substantial socioeconomic implications. Whole-body vibration therapy (WBVT) has shown effectiveness in improving pain and sensorimotor function (e.g., in osteoporosis) in previous studies. However, studies had heterogeneous settings. The aim of this study was to assess the effects of WBVT on pain, function, proprioception, and postural stability in patients with subacute and chronic NLBP.

**Methods:**

A systematic literature search was conducted in the scientific databases PubMed, EMBASE, and PEDro (from inception until 17.05.2023). Only prospective controlled and uncontrolled studies were included. Outcome measures assessed were pain intensity, function (activities of daily living and physical function), proprioception, and postural stability.

**Results:**

A total of 12 original articles (*n* = 821) were included in the analysis. Ten of the studies were randomized controlled trials, one study had a crossover design, and one study had a one-group pre–post study design. The studies compared WBVT vs. no intervention, WBVT vs. basic physical therapy, WBVT vs. core stabilization exercises with and without respiratory resistance, WBVT vs. lumbar extension exercises, and WBVT vs. whole body electromyostimulation training. The treatment approaches varied in terms of duration (2–18 weeks), frequency (2–3 times per week, two applications with a 2-week break), vibration frequency (5–30 Hz), type of exercises (WBVT with or without static or dynamic exercises), and vibration direction (horizontal and vertical). Significant pain reduction was observed in all 10 studies that investigated pain levels. Significant improvement in daily activity function was reported in five of the six studies that investigated daily function, while improvement in physical function was observed in all four studies that investigated physical function. Improvement in proprioception was reported in all three studies that investigated proprioception, and significant improvement in postural stability was observed in four out of six studies that investigated postural stability. No adverse events or side effects related to WBVT were reported.

**Conclusion:**

The majority of the included studies demonstrated significant pain reduction, improvement in physical and daily functioning, and enhanced proprioception. Improvement in postural stability was less consistent. WBVT appears to be a safe and effective treatment modality for subacute and chronic NLBP when used within a multimodal approach. Future research should focus on standardized settings including assessment methods, treatment regimens, frequencies, and intensities.

## Abstract (de)

**Hintergrund:**

Der primäre unspezifische untere Rückenschmerz (NLBP) ist eine häufig auftretende und klinisch bedeutende Erkrankung mit erheblichen sozioökonomischen Auswirkungen. Die Ganzkörper-Vibrationstherapie (WBVT) hat sich bei unterschiedlichen Indikationen zur Verbesserung von Schmerz und Sensomotorik (z. B. Osteoporose) in bis dato sehr heterogenen Studiensettings als wirksam erwiesen. Das Ziel dieser Studie war es, die Auswirkungen der WBVT auf Schmerz, Funktion, Propriozeption und posturale Stabilität bei Patienten mit subakutem und chronischem unspezifischem unterem Rückenschmerz zu bewerten.

**Methoden:**

Es wurde eine systematische Literatursuche in den wissenschaftlichen Datenbanken PubMed, EMBASE und PEDro (von Gründung bis 17.05.2023) durchgeführt. Es wurden nur prospektive kontrollierte und unkontrollierte Studien eingeschlossen. Zielparameter waren Schmerzintensität, Funktion, Propriozeption und posturale Stabilität.

**Ergebnisse:**

In die Analyse wurden insgesamt 12 Originalarbeiten (*n* = 821) eingeschlossen. Davon waren 10 randomisierte kontrollierte Studien, eine Cross-over-Studie und eine Prä-post-Studie ohne Kontrollgruppe. In den Studien erfolgte der Vergleich von WBVT vs. keine Maßnahme, WBVT vs. Bewegungstherapie mit allgemeinen körperlichen Übungen, WBVT vs. rumpfstabilisierende Übungen mit und ohne Atemwegswiderstand, WBVT vs. lumbale Extensionsübungen und WBVT vs. Ganzkörper-Elektromyostimulation. Die Behandlungsansätze variierten in Bezug auf die Dauer (2–18 Wochen), Häufigkeit (2- bis 3‑mal/Woche, 2 Anwendungen mit einer 2‑wöchigen Pause), Vibrationsfrequenz (5–30 Hz), Art der Übungen (WBVT mit oder ohne statische oder dynamische Übungen), und Vibrationsrichtung (horizontal und vertikal). In allen 10 Studien, die die Schmerzintensität untersuchten, wurde eine signifikante Schmerzreduktion festgestellt. In 5 von 6 Studien, die die tägliche Funktion untersuchten, wurde über eine signifikante Verbesserung der täglichen Aktivitäten berichtet, während in allen 4 Studien, die die körperliche Funktion untersuchten, eine signifikante Verbesserung festgestellt wurde. In allen 3 Studien, die die Propriozeption untersuchten, wurde über eine signifikante Verbesserung berichtet. In 4 von 6 Studien, die die posturale Stabilität untersuchten, wurde eine Verbesserung festgestellt. Es wurden keine Nebenwirkungen oder unerwünschten Ereignisse im Zusammenhang mit der WBVT berichtet.

**Schlussfolgerung:**

Die Mehrheit der eingeschlossenen Studien ergab eine signifikante Schmerzreduktion, Verbesserung der körperlichen Funktion sowie eine verbesserte Propriozeption. Die Verbesserung der posturalen Stabilität war weniger konsistent. Daher scheint die Ganzkörper-Vibrationstherapie in Verbindung mit einem multimodalen Ansatz eine sichere und wirksame Behandlung zu sein.

## Introduction

The lifetime prevalence of low back pain (LBP) is estimated to reach up to 84% and the prevalence of chronic low back pain (CLBP) is approximately 23%, with a notable 11–12% of the population experiencing disability due to LBP [3]. After an initial episode of LBP, 44–78% of individuals experience recurrence of pain and 26–37% experience a recurrence of work disability [3].

Non-specific low back pain (NLPB) is a prevalent musculoskeletal condition that has emerged as a significant global public health concern. NLBP does not have a known anatomical, pathological, or neurological origin, and it is typically diagnosed when other potential causes have been ruled out. It is characterized by pain that typically emanates from the lowest rib and extends to the gluteal fold, with the possibility of radiating somatic referred pain into the thigh, extending above the knee [1].

The majority of episodes of NLBP are not associated with significant underlying pathology [2]. However, approximately 10–15% of patients with acute NLBP progress to develop CLPB [2].

Chronic pain, particularly in the context of the healthcare system, poses significant challenges. The chronic condition typically does not improve and consumes a substantial amount of resources [3].

There are numerous potential non-pharmacological therapies for NLBP. Whole-body vibration therapy (WBVT) is usually used for treatment of various musculoskeletal disorders like osteoporosis, osteoarthritis, fibromyalgia, and NLBP [4–7]. The therapeutic effect of WBVT is explained through the tonic vibration reflex [8]. Vibration stimulates the extrafusal muscle fibers, which in turn elicits a stretch reflex of the muscles, representing a general stimulus [9]. This stimulus induced by WBVT is used as a therapeutic effect. The general stimulus of the tonic vibration reflex at 20 Hz during WBVT can lead to relaxation of existing muscle cramps [9]. Additionally, the stretch reflex can also strengthen weak core muscles in this regard [9]. This set of muscles comprises the collection of core muscles encircling the spine and abdominal organs which extend from the diaphragm to the pelvic floor muscles. These muscles collaborate harmoniously to furnish stability to the spinal region [10]. Using an oscillating platform, dynamic and static exercises can be performed in standing, sitting, and lying positions. Different frequencies and amplitudes (0–45 Hz, 0–12 mm) can be adjusted accordingly [11].

A systematic review by Wang et al. (2020) concluded that there is limited evidence suggesting that WBVT is beneficial for NLBP when compared with other forms [7]. However, there is still a lack of standardized protocols for assessments and interventions, as well as a limited number of systematic reviews conducted in the last few years.

Therefore, our study aims to address this gap by summarizing the existing evidence and evaluating the efficacy of WBVT for NLBP. The hypothesis is that therapeutic WBVT is an effective intervention for individuals with NLBP.

## Methods

### Identification and selection of studies

A systematic review was conducted based on the Preferred Reporting Items for Systematic Reviews and Meta-Analysis (PRISMA) guidelines [12].

The search was conducted in the electronic databases PubMed, EMBASE, and PEDro. Studies using the keywords “back pain” or “low back pain” and “whole body vibration” or “WBV” were extracted and considered for inclusion in the study. No filters were applied. The systematic literature search and the assessment of bias risk was independently performed by two researchers, and discrepancies in study selection were resolved through discussion. The search results were screened based on title and abstract. The eligible articles underwent a full-text analysis. Inclusion criteria for the present systematic review comprised studies conducted within primary care settings, specifically emphasizing randomized controlled trials, as guided by the PICOS (population, intervention, comparison, outcomes, and study design) framework [13, 14]. Crossover studies and pre–post studies were also considered for a better review of physiotherapeutic interventions.

### Inclusion and exclusion criteria

Inclusion criteria for the studies were men and women of all ages with subacute and chronic NLBP for at least 6 weeks. Studies were considered suitable for this systematic review if at least one intervention group received WBVT therapy for a minimum duration of 2 weeks with a frequency ranging from one to three sessions per week.

Any quantitative study type of primary and peer-reviewed research that included WBVT as an intervention for NLBP was considered for inclusion. The inclusion criteria were as follows:

- Participants: patients of all ages with NLBP for at least 6 weeks;
- Intervention: WBVT therapy for a minimum duration of 2 weeks;
- Control groups: no WBVT, basic physical therapy, no control group;
- Outcomes: effects on pain, activities of daily living and physical activity, lumbar proprioception, and postural stability;
- Study design: prospective, controlled, and uncontrolled studies, crossover design;
- Language limitations: published in English or German.

Retrospective trials, case reports, reviews, letters, editorials, commentaries, and conference papers were excluded.

Studies examining patients with work-related vibration, such as truck drivers, were excluded. Likewise, studies analyzing subjects without LBP who received WBVT therapy were excluded.

### Data collection and analysis

The following data were collected: author, publication year, country, study design, sample size, dropout rate, and authors’ conclusions. Patient characteristics included mean age and gender. Intervention characteristics encompassed the use of the vibration device, settings such as hertz and amplitude, type of exercises (static or dynamic) on the vibration device, treatment duration, and follow-up period. We also recorded the treatment provided to the control group. The primary outcome measures of this review were pain assessed using the visual analog scale (VAS) and the numerical rating scale (NRS), the Roland Morris Disability Scale (RMQ), and the Oswestry Disability Index (ODI). Secondary outcomes examined included lumbar proprioception and postural stability.

The outcome data were reported as median value with standard deviation. The significance level between study groups and the significance level in the study group before and after therapy were indicated using the *p*-value. A few studies provided the effect size according to Cohen and a between-group difference with a 95% confidence interval.

The assessment of bias risk for randomized studies was conducted using the PEDro criterion.

## Results

### Literature search

**Fig. 1:**
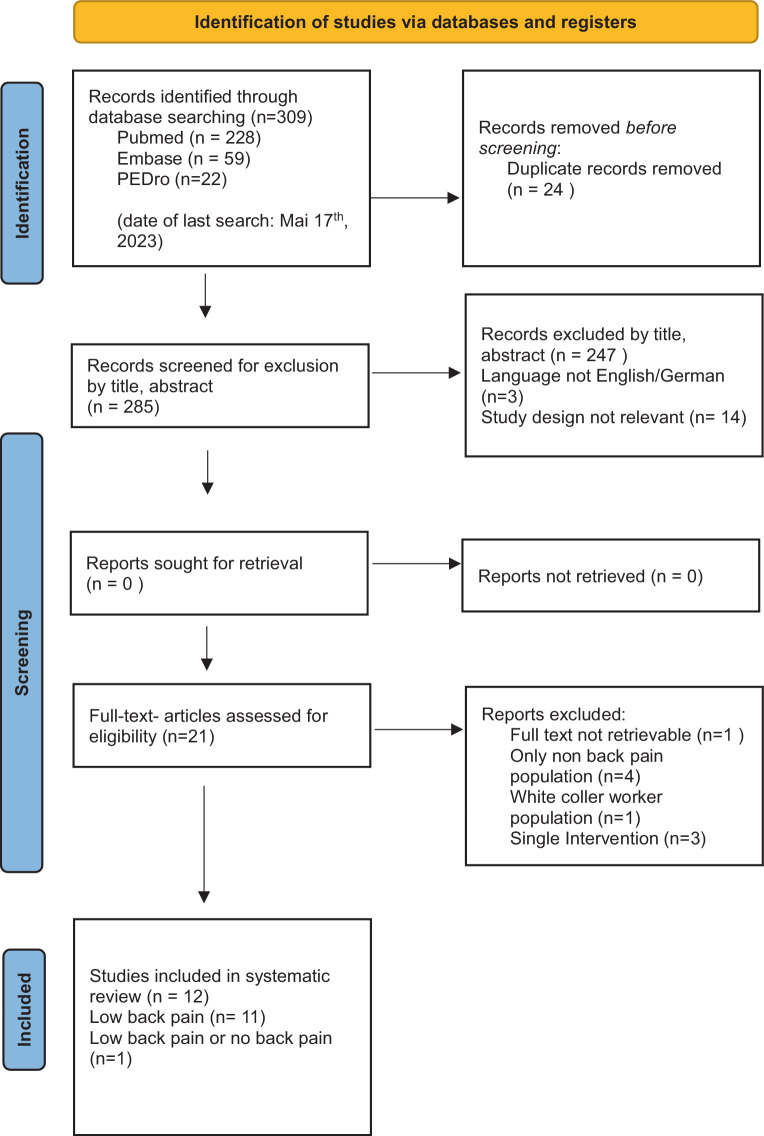
Identification of studies

A total of 309 studies published until May 17, 2023 were identified and screened for eligibility based on their titles and abstracts as depicted in Fig. 1. After removing duplicates, 254 studies were rejected as non-eligible; 21 studies were selected for full-text analysis and 12 studies met the inclusion criteria [15–26]. Among these, there were 10 randomized controlled trials [15, 16, 18–24, 26], one crossover study [25], and one single-group pre- and post-test design [17].

The risk of bias analysis is summarized in Table 1. The PEDro scale ranged from 4–9 points, showing that the articles were of fair to good methodological quality. Nine studies demonstrated good quality, while two studies had fair quality (Table 1).

**Table 1:** Level of evidence for the included studies, analyzed with the PEDro scale

Clinical trail	Items on the PEDro scale											Total score	Level of quality
	1	2	3	4	5	6	7	8	9	10	11		
Wang et al. (2019) [15]	1	1	1	1	0	0	1	1	1	1	1	9	Good
Wegener et al. (2019) [16]	1	1	0	1	0	0	0	0	0	1	1	5	Fair
Kaeding et al. (2017) [17]	1	1	0	1	0	0	1	1	1	1	1	8	Good
Pozo-Cruz et al. (2011) [18]	1	1	0	1	0	0	1	1	1	1	1	8	Good
Rittweger et al. (2002) [19]	1	1	0	0	0	0	0	0	0	1	1	4	Fair
Karacay et al. (2022) [20]	1	1	0	1	0	0	1	1	1	1	1	8	Good
Yang et al. (2015) [21]	1	1	0	1	0	0	0	1	1	1	1	7	Good
Zheng et al. (2019) [22]	One-group intervention											–	–
Micke et al. (2021) [23]	1	1	0	1	0	0	1	1	1	1	1	8	Good
Kim et al. (2018) [24]	1	1	0	1	1	0	0	1	1	1	1	8	Good
Sajadi et al. (2019) [25]	1	1	0	1	0	0	0	1	1	1	1	7	Good
Park et al. (2022) [26]	1	1	0	1	1	0	0	1	0	1	1	7	Good

The included studies were heterogeneous, both in terms of treatment protocols and in terms of outcome parameters. Pooling of data for meta-analysis was not possible. Therefore, we were limited to describing the included studies.

### Study characteristics

The key characteristics of the studies are summarized in Table 2.

**Table 2:** Study characteristics and investigated outcomes with authors’ conclusion

Study	Population	Intervention	Outcome	Results/authors’ conclusion
Wang et al. (2019) [15]	Single-blind randomized controlled trial			
	89 patients with NCLBP	12 weeks 3 times per week 5 min warm up, 15 min exercises (6 dynamic and static movements: squat, kneeling, bridge, bridge with leg lift, bridge and knee flex, and back release) 5 min cool down	VAS ODI Joint position sense in flexion/extension Con-Trex isokinetic dynamometer SF36 Global perceived effect Adverse events	“The study provided evidence that whole-body vibration exercise could offer greater advantages compared to general exercise in terms of pain relief and improvement of functional disability in patients suffering from non-specific chronic low back pain.” [15]
	Intervention group: 45 patients Mean age in years (SD): 21.64 (3.01) Gender m/f: 34/11 Dropout: 4	BodyGreen 9 Hz: WBV		
	Control group: 44 patients Mean age in years (SD): 22.02 (4.59) Gender m/f: 31/13 Dropout: 1	Without vibration		
Wegener et al. (2019) [16]	Two-step prospective randomized parallel trial			
	115 patients with and without non-specific chronic back pain	–	–	“In the study setting, the MFT-S3-Check did not detect a significant difference in postural stability between the group of individuals with back pain and the group without back pain. However, it was observed that postural stability improved after the implementation of treatment.” [16]
	50 patients no back pain Mean age in years (SD): 61.2 (8.6) Gender m/f: 20/30	–	MFT S3-Check (muscle-mediated spine stability): Trend Sport Trading GmbH, Großhöflein, Austria STI, SMI, SI	
	65 patients with NSCBP Mean age in years (SD): 61.6 (7.9) Gender m/f: 20/45 Dropout: 11	18 weeks 3 × 6 weeks with increasing intensity and increasing time Twice a week 5 defined trunk stability exercises	MFT S3-Check ODI SF-36 physical/mental summary HADS NASS/back pain & neurological Symptoms	
	Intervention group (WBV) Intervention group: 22 patients	Galileo® 5–20 Hz		
	Control group (CPT) Control group: 22 patients	Weights and TheraBand®		
Kaeding et al. (2017) [17]	Randomized and controlled study			
	41 patients with chronic low back pain	12 weeks	RMQ ODI (%) Static posturography: Leonardo Mechanograph SF 36 (physical, mental health) WAI Freiburger activity questionnaire, Isokinetic performance of the musculature of the trunk Sick leave	“WBV training appears to be a beneficial, secure, and appropriate intervention for individuals with chronic low-back pain who engage in seated work.” [17]
	Intervention group: 21 patients Mean age in years (SD): 46.4 (9.3) Gender m/f: 14/7 Dropout: 1	Galileo® 10–30 Hz (increasing during the intervention), 1.5–3.5 mm, 2.5 times per week 15 min 5 sets with a duration of 60–120 s Basic position on the device:		
	Control group: 20 patients Mean age in years (SD): 45.5 (9.1) Gender m/f: 14/6 Dropout: 1	No treatment		
Pozo-Cruz et al. (2011) [18]	Single-blind randomized controlled trial			
	50 patients with NCLBP	12 weeks	RMQ (points) VAS ODI (%) Postural stability test: BIODex balance system PSTAntPost, PSTMedLat HRQoL T6MWT PILE Peripheral vibration sensibility: Vibratron II device	“A twelve-week program of low-frequency vibrating board therapy is viable and potentially offers a new approach to physical therapy for individuals with non-specific low back pain.” [18]
	Intervention group: 25 patients Mean age in years (SD): 58.71 (4.59) Gender m/f in %: 28/72	Galileo® 20 Hz Twice a week with increasing time per series (60–360 s), decreasing repetition (6-1) Basic static position with 120° knee angle		
	Control group: 25 patients Mean age in years (SD): 59.53 (5.47) Gender m/f in %: 26/74 Dropout: 1	No treatment		
Rittweger et al. (2002) [19]	Randomized and controlled study			
	60 patients with CLBP	12 weeks, 2nd follow-up after 6 months Twice a week first 6 weeks Once a week last 6 weeks	P‑VAS PDI Isometric lumbar extension torque: Le Mark 1 lumbar extension machine ROM lumbar flexion and extension: Le Mark 1 lumbar extension machine	“The existing data suggest that diminished lumbar muscle strength is likely not the sole underlying factor responsible for chronic lower back pain. Various forms of exercise therapy appear to produce similar outcomes. Interestingly, well-regulated vibration may serve as a solution rather than a trigger for lower back pain.” [19]
	Intervention group: 30 patients Mean age in years (SD): 54.1 (3.4) Gender: 15/15 Dropout: 5	Galileo® 18 Hz, 6 mm 2 min warmup Max. 7 min intervention: slow movements of the hips, waist with bending and rotation		
	Control group: 30 patients Mean age in years (SD): 49.8 (6.6) Gender: 15/15 Dropout: 5	LE Mark 1 Isodynamic lumbar extension exercises 1 min warmup with lumbar extension Repetitive contraction cycles at a constant speed with a torque corresponding to 50% of the baseline maximum isometric values		
Karacay et al. (2022) [20]	Randomized controlled trial			
	84 patients with NCLBP	8 weeks, 2nd follow-up after 3 months 3 times a week	VAS RMQ PILE IMS: CybexTM Human Norm 350	“In the treatment of NLBP, WBV and core stabilization exercise (CSE) appear to be effective in reducing pain and improving functionality. While there was a significant improvement in muscle strength and functional work performance in all three groups, the WBVE group and CSE group showed greater improvements compared to the control group (CG).” [20]
	Intervention group: WBV 28 patients Mean age in years (SD): 43.3 (9.2) Gender m/f: 5/20 Dropout: 3	Power plate®: 25 Hz, 2 mm 5 min warmup 3 different static positions, 30–60 s per position (squat, bridge, push-up) + classic lumbar home exercises		
	Control group 1: CSE 28 patients Mean age in years (SD): 47.2 (8.0) Gender m/f: 2/23 Dropout: 3	5 min warmup, 30 min core stabilization exercises + classic lumbar home exercises		
	Control group 2: CG 28 patients Mean age in years (SD): 43.6 (9.4) Gender m/f: 5/19 Dropout: 4	Classic lumbar home exercises		
Yang et al. (2015) [21]	Randomized controlled trial			
	40 patients with LBP No dropout	3 weeks 3 times a week	VAS ODI FI: Tetrax KA: 3D tomography LA: 3D tomography	“WBV can be recommended as a therapeutic intervention for improving balance ability and alleviating pain in patients with chronic lower back pain.” [21]
	Intervention group: 20 patients Mean age in years: 32.80 Gender m/f: 12/8	Galileo® 18 Hz 5 min of WBV Static standing position 25 min of lumbar stability training with pressure biofeedback		
	Control group: 20 patients Mean age in years: 30.95 Gender m/f: 9/11	30 min of lumbar stability training with pressure biofeedback		
Zheng et al. (2019) [22]	One group pre-test, post-test			
	43 patients with NCLBP Mean age in years (SD): 21.6 (3) Gender m/f: 32/10 Dropout *n* =1	12 weeks BodyGreen: 9 Hz, 2 mm 3 times a week 5 min warmup, 18 min training (6 postures: squat, kneeling, bridge, bridge with leg lift, bridge and knee flex, and back release) 5 min cool down	VAS Lumbar joint position sense: Con-Trex Multi Joint system (Switzerland)	“After a 12-week WBV exercise, lumbar flexion and extension proprioception, as measured by joint position sense, showed significant improvement, and pain levels were significantly reduced in patients with NSLBP. However, it was observed that patients with already good flexion proprioceptive ability had limited enhancement in proprioception.” [22]
Micke et al. (2021) [23]	Three-armed randomized controlled trial			
	240 chronic NLBP	12 weeks	NRS (diary during the last four weeks of intervention) Trunk extension strength: BackCheck	“WB-EMS, WBV, and conventional training (CT) have demonstrated comparable effectiveness in improving maximum power input (MPI) and trunk strength. However, the training volume of WB-EMS was found to be 43 or 62% lower compared to CT and WBV, respectively.” [23] *Bias:* Change of intake of analgesics Start of additional treatment during the intervention (26 participants)
	Intervention group: WBV 80 patients Mean age in years: 54.30 Gender m/f: 30/50 Dropout: 10	Wellengang: 5–10 Hz, 9 mm Twice a week 15 min 5 exercises dynamic and static: dynamic cable squats, squats with arm extension, calf raises, static squats with arm movement, and static cable squats with calf raises		
	Control group 1: WB-EMS 80 patients Mean age in years: 54.10 Gender: 30/50 Dropout: 7	WB EMS Once a week 20 min 6 trunk-specific exercises		
	Control group 2: CT 80 patients Mean age in years: 58.3 Gender m/f: 32/48 Dropout: 10	Once a week 45 min, 15 min warmup, 30 min circuit training with static and dynamic exercises		
Kim et al. (2018) [24]	Randomized controlled trial			
	28 patients with CLBP Dropout: 4	12 weeks, 2nd follow-up after 4 weeks 3 times a week 30 min 5 min warmup and cool down 20 min static posture	VAS ODI Standing balance: Biodex Stability System (Biodex Medical Systems). Muscle strength lumbar: isokinetic dynamometer Thickness TrA, MF: ultrasound	“HVE is shown to be equally effective as VVE in reducing pain, strengthening the lumbar muscles, and improving balance and functional abilities in patients with CLBP. Vibrational exercise has the ability to increase muscle strength without inducing muscle hypertrophy.” [24]
	Group A: HVE 14 patients Mean age in years (SD): 55.1 (11.2) Gender m/f: 4/10	Horizontal vibration exercise 3–5 Hz 1–48 mm 3 times a week Vibration in anteroposterior and in mediolateral directions		
	Group B: VVE 14 patients Mean age in years (SD): 53.7 (12.1) Gender m/f: 3/11	Vertical vibration exercise 28–34 Hz, 2.5–5 mm		
Sajadi et al. (2019) [25]	Randomized crossover trial			
	24 patients with CLBP Mean age in years (SD): 25.2 (2.6) Gender m/f: 12/12 No dropout	Two sessions with 2 weeks of rest between them Power Plate®: LF (30 Hz), HF (50 Hz), 2.5 mm Semi-squat position 5 × 1 min with 1 min rest	VAS RE recorded in neutral, 30% and 60% of its maximum available range before and 5 min after WBV: electrogoniometer	“In this study, the method of WBV used, low-frequency WBV, appears to lead to greater improvement in the accuracy of lumbopelvic repositioning compared to high-frequency WBV.” [25]
	Group A: 12 patients: First session with LF WBV, second session with HF WBV			
	Group B: 12 patients: first session with HF WBV, second session with LF WBV			
Park et al. (2022) [26]	Randomized controlled trial			
	48 patients with LBP	5 weeks 60 min, 3 times a week 6 dynamic exercises Per exercise: 5 repetitions in a set, 10 s for each set, and 5 sets total	4‑item visual analogue scale (QVAS) RMQ Static balance ability: Wii Balance board (Nintendo Kyoto, Japan) Center of pressure velocity Center of pressure Length Center of pressure area FABQ-PA, FABQ‑W, FVC, FEV1, FEV1/FVC MVV MEP MIV	“The implementation of a stabilization exercise program that incorporates respiratory resistance and whole-body vibration, tailored to the specific objectives of the intervention, appears to be an effective exercise regimen for individuals with lumbar instability.” [26]
	Intervention group: SERW 16 patients Mean age in years (SD): 30.93 (4.70) Gender m/f: 8/6 Dropout: 2	SW-VH11, Wonju, Korea, 30 Hz Stabilization exercise + WBV + respiratory resistance (SERW)		
	Control group 1: SER 16 patients Mean age in years (SD): 31.07 (6.82) Gender m/f: 9/5 Dropout: 2	Stabilization exercise + respiratory resistance (SER)		
	Control group 2: SE 16 patients Mean age in years (SD): 30.29 (5.34) Gender m/f6/8 Dropout: 1	Stabilization exercise (SE)		

*CLBP* chronic low back pain, *NSCBP* non-specific chronic back pain, *VAS* visual analog scale, *ODI* Oswestry Disability Index, *SF36* Short Form 36 Health Survey, *WBV* whole-body vibration, *SD* standard deviation, *MFT S3* muscle-mediated spine stability, *STI* stability index, *SMI* sensorimotor index, *SI* symmetry index, *HADS* Hospital Anxiety and Depression Scale, *NASS* North American Spine Society, *CPT* classic physiotherapy, *sEMG* surface electromyography, *ES* erector spinae, *MF* multifidus, *TfA/IO* transversus abdominis/internal oblique, *RA* rectus abdominis, *WAI* work ability questionnaire, *HRQoL* health-related quality of life, *PSTAntPost* anterior posterior score, *PSTMedLat* medial lateral score, *6 MWT* six-minute walk test, *PILE* progressive isointertial lifting evaluation, *PDI* Pain Disability Index, *P‑VAS* pain visual analog scale, *ROM* range of motion, *IMS* isokinetic muscle strengths, *CSE* core stabilization exercises, *CG* control group, *FI* fall index, *KA* kyphotic angle, *LA* lordosis angle, *NRS* numeric rating scale, *WB EMS* whole body electromyostimulation, *WBV* whole-body vibration, *CT* circuit training, *HF* high frequency, *LF* low frequency, *HVE* horizontal vibration exercise, *VVE* vertical vibration exercise, *RE* reposition error, *OE* obliquus externus, *FABQ-PA/W* fear avoidance belief questionnaire-physical activity/work, *FVC* forced vital capacity, *FEV1* forced expiratory volume in one second, *MVV* maximum voluntary ventilation, *MEP* maximum expiratory pressure, *MIV* maximum inspiratory pressure

### Participants

A total of 821 study participants were included. The average age of the subjects ranged from 21.6 to 61.6 years. Among them, 658 participants had CLBP, 48 participants had subacute LBP, 65 participants had chronic back pain (CBP), and 50 participants had no back pain. 21 participants with CBP dropped out before receiving vibration therapy, and all 50 patients with no back pain did not receive any intervention.

### Intervention

Of the 381 participants receiving WBVT, 317 received WBVT as a standalone therapy [15–19, 22–25], 20 participants received WBVT in combination with lumbar stabilization training with biofeedback [21], 16 participants with subacute LBP received WBVT with respiratory resistance therapy and stabilizing exercises (SERW) [26], and 28 participants received WBVT along with home exercises [20].

The implementation of WBVT differed among the studies. Some studies only included dynamic exercises [16, 19], while others combined dynamic and static movement exercises [15, 23]. On the other hand, some studies focused on holding static postural exercises in various positions [20, 22, 26], or only adopted a basic position with slight knee flexion [17, 18, 21, 24, 25].

The control groups received different treatment approaches like no treatment [17], basic physical exercises [15, 23], trunk-stabilizing exercises with [21] and without biofeedback [16], trunk-stabilizing exercises along with home exercises [20], exercises solely at home [20], stabilizing exercises with respiratory resistance (SER) and without respiratory resistance (SE) [26], whole-body electromyostimulation (WB EMS) therapy with trunk-specific exercises [23], and isodynamic lumbar extension exercises [19].

Not all studies compared WBVT with non-WBVT. One crossover study compared low-frequency WBVT (LF) at 30 Hz with high-frequency WBVT (HF) at 50 Hz [25]. Another study investigated differences between horizontal and vertical WBVT [23]. A one-group pre-test and post-test study examined the impact of WBVT in a single group [22].

Different vibration platforms were utilized: BodyGreen® [B.Green Technology Co., Ltd., Xiushui Township, Changhua County, Taiwan (R.O.C.)], [15, 22]. Galileo® [16–19, 21], Power Plate® [20, 25], Wellengang platform® [23], and sW–VH11 platform® [26].

Different frequencies of WBVT were applied in the included studies. Two studies had increasing frequencies over the course of the study, ranging from 5 to 20 Hz [16] and from 10 to 30 Hz [17]. Frequencies of 9 Hz [15, 22], 10 Hz [23], 18 Hz [19, 21], 20 Hz [18], 25 Hz [20], and 30 Hz [26] were examined.

Different studies with varying treatment durations were included in this review, ranging from 2 weeks to 18 weeks.

Three studies examined follow-up periods after completion of therapy, with durations of 6 months [19], 3 months [20], and 1 month [24].

### Outcome

#### Primary evaluated outcomes: pain intensity, impact on activities of daily living and physical function

The primary outcomes in this systematic review involve evaluation of the effects of WBVT, with a focus on examining the changes in pain intensity and functional impairment. Overall, ten out of the 12 studies assessed pain intensity before and after the intervention using the visual analog scale (VAS), the numeric rating scale (NRS), or the categorical quantitative VAS (QVAS) [15, 18–26]. The impact of back pain on activities of daily living was assessed using the Oswestry Disability Index (ODI) in six studies [15–18, 21, 24]. To assess physical function impairment due to back pain, four out of the 12 studies investigated the Roland Morris Disability Score (RMQ; Table 3).

**Table 3:** Primary outcome, VAS, NRS, QVAS, ODI, RMQ

Author	Time period	Tests	Groups	Initial	Follow up		*P*-value	Effect size	Between-group difference (95% CI)
Wang et al. [15]	12 weeks	VAS adjusted (ITT)	Intervention (*n* = 45)	4.39 (4.16–4.61)	2.87 (2.53–3.21)		< 0.001^e^	−1.04	−1 (−1.22, −0.78)
			Control (*n* = 44)	4.05 (3.83–4.29)	3.87 (3.53–4.21)				
		ODI adjusted (ITT)	Intervention (*n* = 45)	32.46 (30.22–34.71)	19.46 (17.69–21.24)		< 0.001^e^	−0.75	−3.81 (−4.98, −2.63)
			Control (*n* = 44)	32.18 (29.91–34.5)	23.27 (21.47–25.07)				
		VAS unadjusted	Intervention (*n* = 45)	–	–		< 0.001^e^	–	0.89 (−1.41 to −0.36)
			Control 0 (*n* = 44)	–	–				
		ODI unadjusted	Intervention (*n* = 45)	–	–		< 0.001^e^	–	−3.81 (−6.68 to −0.92)
			Control (*n* = 44)	–	–				
		VAS	Global perceived effect and minimal clinically important difference						
			Intervention (*n* = 45)	Control group (*n* = 44)	–		0.001^e^	–	4.7 (1.79 to 12.33)^c^
		Benefit	23 (51.1)	8 (18.2)	–		–	–	–
		No benefit	22 (48.9)	36 (81.8)	–		–	–	–
		ODI	Global perceived effect and minimal clinically important difference						
			Intervention (*n* = 45)	Control group (*n* = 44)	–		0.013^e^	–	3.01 (1.24–7.31)^c^
		Benefit	33 (73.3)	21 (47.7)	–		–	–	–
		No benefit	12 (26.7)	23 (52.3)	–		–	–	–
Wegener et al. [16]	18 weeks	ODI	–				0.304^e^	–	–
			Intervention (*n* = 22)	18.1 (12.0)	17.1 (11.9)		0.185^f^	–	–
			Control (*n* = 22)	20.7 (11.4)	16.6 (12.3)		0.876^f^	–	–
Kaeding et al. [17]	12 weeks	ODI PP population	–	–	–		0.002^d,e^ < 0.001^a,e^	–	2.7 (1.3–4.2) (PP)
			Intervention (*n* = 21)	17.2 (9.2)	12.3 (7.4)		–	–	4.5 (6.6)^b^
			Control (*n* = 20)	15.7 (7.1)	17.3 (6.8)		–	–	−1.2 (3.2)^b^
		RMQ ITT population	–	–	–		0.043^d,e^ 0.027^a,e^	0.69	1.8 (0.2–3.4) (PP)
			Intervention (21)	4.0 (3.8)	2.3 (2.9)		–	–	1.5 (2.6)^b^
			Control (20)	3.5 (2.3)	4.0 (2.4)		–	–	−0.3 (2.6)^b^
		RMQ PP population	–	–	–		0.072^d,e^ 0.008^a,e^	–	–
			Intervention (19)	4.0 (3.8)	1.7 (1.8)		–	–	–
			Control (16)	3.5 (2.3)	3.6 (2.5)		–	–	–
Pozo-Cruz et al. [18]	12 weeks	VAS	–	–	–		0.006 (24.13%)^e^	−0.85	−9.40 (2.94 to 16.05)^g^
			Intervention (25)	38.36 (15.85)	29.00 (13.02)		–	–	–
			Control (25)	39.65 (13.26)	39.68 (14.77)		–	–	–
		ODI	–	–	–		0.013 (25.15%)^e^	−0.72	−6.3 (−13.7 to −1.70)^g^
			Intervention (25)	26.50 (17.00)	20.28 (10.89)		–	–	–
			Control (25)	29.16 (15.78)	29.24 (15.64)		–	–	–
		RMQ	–	–	–		0.001 (9.31%)^e^	−1.01	−1.12 (−2.42 to 0.96)^g^
			Intervention (25)	11.63 (8.35)	10.47 (8.68)		–	–	–
			Control (25)	12.44 (4.46)	12.40 (4.50)		–	–	–
Rittweger et al. [19]	12 weeks	VAS	–	–	–		> 0.2^e^	–	–
			Intervention (25)	4.16 (1.86)	1.40 (1.83)		< 0.001^f^	–	–
			Control (25)	4.52 (2.21)	1.20 (1.76)		< 0.001^f^	–	–
Karacay et al. [20]	8 weeks Second follow-up after 3 months	VAS in rest/VAS in activity (PP)	–	–	–		0.023^e^	–	–
			Intervention (25)	3.0 (1.5)/5.5 (1.5)	–		< 0.001^f^	–	–
			Control (25)	3.2 (1.9)/5.5 (1.7)	–		< 0.001^f^	–	–
			Control 2 (24)	3.7 (1.9)/6.0 (1.7)	–		0.214^f^	–	–
		RMQ (PP)	–	–	–	Follow up after 3 months	0.73^e^	–	Difference pretreatment–posttreatment 3rd month
			Intervention (25)	4.82 (3.97)/	1.86 (2.63)	1.47 (1.90)	< 0.001^f^	–	3.34 (3.73)^b^
			Control (25)	4.44 (4.9)	2.44 (3.16)	1.80 (2.02)	< 0.001^f^	–	2.64 (3.69)^b^
			Control 2 (24)	6.30 (6.03)	4.91 (4.28)	5.26 (5.41)	0.427^f^	–	1.04 (6.57)^b^
Yang et al. [21]	3 weeks	VAS	–	–	–		< 0.005^e^	–	–
			Intervention (*n* = 20)	5.60 (1.60)	2.70 (1.26)		< 0.001^f^	–	–
			Control (*n* = 20)	5.25 (1.12)	3.50 (1.12)		< 0.001^f^	–	–
		ODI	–	–	–		Not significant	–	–
			Intervention (*n* = 20)	17. 85 (11.09)	12.45 (6.06)		< 0.001^f^	–	–
			Control (*n* = 20)	15.30 (7.57)	12.80 (6.67)		< 0.001^f^	–	–
Zheng et al. [22]	12 weeks	VAS	Intervention (42)	4.62 (1.23)	3.0 (1.38)		0.0001^f^	0.96	−28.3 (47.9)^b^
Micke et al. [23]	12 weeks	NRS	–	–	–		0.934^e^	–	–
			Intervention (*n* = 80)	2.94 (1.51)	–		< 0.001^f^	–	30.3 (39.3%)^b^
			Control (*n* = 80)	3.08 (1.89)	–		< 0.001^f^	–	29.7 (39.1%)^b^
			Control 2 (*n* = 80)	3.10 (1.57)	–		< 0.001^f^	–	30.5 (39.6%)^b^
Kim et al. [24]	4 weeks Second follow-up after 1 month	VAS	–	–	–	Posttreatment 1 month	0.929^e^	–	–
			Horizontal (*n* = 14)	4.30 (1.50)	2.00 (0.90)	2.60 (1.00)	0.001^f^	–	–
			Vertical (*n* = 14)	4.90 (1.90)	2.80 (1.30)	3.10 (1.50)	0.001^f^	–	–
		ODI	–	–	–	–	0.595^e^	–	–
			Horizontal (*n* = 14)	21.57 (4.11)	14.57 (3.67)	14.71 (5.47)	0.001^f^	–	–
			Vertical (*n* = 14)	22.36 (6.76)	16.21 (4.02)	17.43 (5.42)	0.001^f^	–	–
Sajadi et al. [25]	2 weeks	VAS	–	–	–		No significance	–	–
			LF WBV (*n* = 24)	45.8 (10.2)	36.6 (7.8)		0.000^f^	–	9.22^b^
			HF WBV (*n* = 24)	45.8 (10.2)	34.7 (8.7)		0.000^f^	–	11.05^b^
		ODI	LF WBV (*n* = 24)	12.46 (8.51)	–		–	–	–
			HF WBV (*n* = 24)	12.46 (8.51)	–		–	–	–
Park et al. [26]	2 weeks	QVAS	–	–	–		–	–	Post–Pre
			SERW (*n* = 16)	6.41 (0.43)	4.59 (0.39)		0.000^f^	–	−1.82 (0.58)^b^
			SER (*n* = 16)	6.45 (0.44)	4.66 (0.409)		0.000^f^	–	−1.79 (0.43)^b^
			SE (*n* = 16)	6.46 (0.46)	5.96 (0.87)		0.035^f^	–	−0.5 (0.79)^b^
		RMQ	SERW (*n* = 16)	21.29 (1.59)	10.29 (1.27)		0.000^f^	–	−11 (0.96)^b^
			SER (*n* = 16)	21.43 (1.60)	10.14 (1.17)		0.000^f^	–	−11.29 (1.14)^b^
			SE (*n* = 16)	21.14 (1.61)	13.07 (1.44)		0.000^f^	–	−8.07 (1.86)^b^

*ITT* intention to treat, *PP* per protocol, *VAS* visual analogue scale, *ODI* Oswestry Disability Index, *RMD* Roland Morris Disability Index, *NRS* numeric rating scale, *QVAS* quadruple visual analogue scale

^a^analysis of covariance

^b^change

^c^odds ratio

^d^*t*-test

^e^between-group difference

^f^ingroup difference

^g^treatment effect mean (95% confidence interval)

#### Secondarily evaluated outcomes: trunk proprioception, postural stability

Three studies included in this review examined trunk proprioception, investigating joint position sense in flexion and extension using the Con-Trex Multi Joint system® [15, 22]. The repositioning error at 0%, 30%, and 60% of the maximum range of motion using an electrogoniometer for both low-frequency (LF) and high-frequency (HF) vibration therapy was examined [25].

Five out of the 12 studies investigated postural stability. [16–18, 21, 24]. One study used the MFT S3 Checks that determine the stability index (STI), sensorimotor index (SMI), and symmetry index (SI) in both standing and sitting positions [16]. Another study measured the fall index (FI) over time using posturography (Tetrax®) [21]. One study examined postural control using the Leonardo Mechanograph®, assessing standard deviations in the anterior–posterior and medio–lateral directions [17]. Two studies also investigated postural stability using the anterior–posterior stability index (PSTAntPost/AP) and medial–lateral stability index (PSTMedLat/ML) with the Biodex balance system™ [18, 24]. One study measured static balance ability using a Wii and calculated the center of pressure (CoP), velocity, length, and area ([26]; Table 4).

**Table 4:** Secondary outcome: proprioception and postural stability

Author	Tests	Groups	Initial		Follow-up		*p*-value	Effect size	Between-group difference (95% CI)
Wang et al. [15]	Joint position sense Flexion Adjusted	Intervention (*n* = 45)	3.55 (2.82, 4.28)		1.91 (1.36, 2.44)		0.005^a^	−1.14	1.76 (−2.11, −1.4)
		Control (*n* = 44)	3.96 (3.22, 4.7)		3.67 (3.12, 4.21)				
	Joint position sense Extension Adjusted	Intervention (*n* = 45)	2.96 (2.23, 3.64)		1.66 (1.17, 2.15)		0.036^a^	−0.78	−1.1 (−1.42, −0.77)
		Control (*n* = 44)	3.06 (2.37, 3.74)		2.76 (2.26, 3.26)				
	Joint position sense Flexion unadjusted	Intervention (*n* = 45)	–		–		0.003^a^	–	–
		Control (*n* = 44)	–		–				
	Joint position sense Extension unadjusted	Intervention (*n* = 45)	–		–		0.016^a^	–	–
		Control (*n* = 44)	–		–				
Wegener et al. [16]	STI standing (PP)	NBP (*n* = 50)	5.1 (1.0)		–		–	–	–
		BP (*n* = 65)	5.0 (1.3)		–		0.885^a^	–	–
		Intervention group (*n* = 17)	5.0 (1.0)		4.4 (1.1)		0.052^b^	–	–
		Control group (*n* = 16)	5.6 (1.5)		4.8 (1.4)		0.012^b^	–	–
	STI seated (PP)	NBP (*n* = 50)	3.6 (2.0)		–		–	–	–
		BP (*n* = 65)	4.0 (2.4)		–		0.516^a^	–	–
		Intervention group (*n* = 17)	3.9 (2.1)		2.9 (2.4)		0.073^b^	–	–
		Control group (*n* = 16)	4.9 (2.9)		2.6 (2.3)		0.015^b^	–	–
	SMI standing (PP)	NBP (*n* = 50)	4.1 (1.2)		–		–	–	–
		BP (*n* = 65)	4.3 (1.3)		–		0.493^a^	–	–
		Intervention group (*n* = 17)	4.0 (1.2)		3.4 (1.2)		0.080^b^	–	–
		Control group (*n* = 16)	5.0 (1.2)		4.0 (1.2)		0.006^b^	–	–
	SMI seated (PP)	NBP (*n* = 50)	2.9 (1.7)		–		–	–	–
		BP (*n* = 65)	3.4 (2.3)		–		0.217^a^	–	–
		Intervention group (*n* = 17)	2.9 (1.9)		2.0 (1.6)		0.065^b^	–	–
		Control group (*n* = 16)	4.1 (2.7)		2.0 (1.8)		0.004^b^	–	–
	SI standing right (PP)	NBP (*n* = 50)	48.5 (12.6)		–		–	–	–
		BP (*n* = 65)	49.9 (9.7)		–		0.718^a^	–	–
		Intervention group (*n* = 22)	52.2 (13.0)		47.8 (14.4)		0.245^b^	–	–
		Control group (*n* = 22)	49.3 (7.5)		50.8 (10.3)		0.594^b^	–	–
	STI standing left (PP)	NBP (*n* = 50)	51.6 (13.1)		–		–	–	–
		BP (*n* = 65)	50.1 (9.7)		52.2 (14.4)		0.718^a^	–	–
		Intervention group (*n* = 22)	47.8 (13.0)		52.2 (14.4)		0.245^b^	–	–
		Control group (*n* = 22)	50.7 (7.5)		49.3 (10.3)		0.594^b^	–	–
	SI seated right (PP)	NBP (*n* = 50)	57.2 (16.2)		–		–	–	–
		BP (*n* = 65)	50.7 (15.4)		–		0.773^a^	–	–
		Intervention group (*n* = 22)	48.3 (22.0)		51.7 (21.9)		0.865^b^	–	–
		Control group (*n* = 22)	55.5 (15.1)		54.1 (17.5)		0.754^b^	–	–
	STI seated left (PP)	NBP (*n* = 50)	42.8 (16.2)		–		–	–	–
		BP (*n* = 65)	49.3 (15.4)		–		0.773^a^	–	–
		Intervention group (*n* = 22)	51.7 (22.0)		51.7 (21.9)		0.865^b^	–	–
		Control group (*n* = 22)	44.5 (15.1)		45.9 (17.5)		0.754^b^	–	–
Kaeding et al. [17]	Static posturography: there were no significant differences in the tests with eyes open or with eyes closed in any of the measured parameters between the groups after 3 months of EBV training								
Pozo-Cruz et al. [18]	PSTAntPost	Intervention (*n* = 25)	0.52 (0.22)		0.41(0.95)		0.031^a^ (ANOVA)	−3.74	−0.11 (−0.22 to 0.00)^d^
		Control (*n* = 24)	0.57 (0.40)		0.57 (0.40)				
	PSTMedLat	Intervention (*n* = 25)	0.33 (0.17)		0.30 (0.21)		0.422^a^	−0.20	−0.03 (−0.13 to −0.05)^d^
		Control (*n* = 24)	0.47 (0.36)		0.47 (0.37)				
Yang et al. [21]	Fall index	–	–		–		< 0.05^a^	–	–
		Intervention (*n* = 20)	30.59 (14.97)		12.80 (10.39)		< 0.001^b^	–	–
		Control group (*n* = 20)	23.40 (12.73)		21.69 (12.68)		–	–	–
Zheng et al. [22]	Flexion angle deviation	Intervention group (42)	3.65 (2.26)		1.90 (1.07)		0.0001^b^	−26.4^e^ (57.7)	0.75^f^
	Extension angle deviation	Intervention group (42)	3.06 (1.85)		1.61 (0.75)		0.0001^b^	−35.9 (27.9)^e^	0.83^f^
Kim et al. [24]	Anterior/posterior	–				Follow up after 1 month	0.647^a^	–	–
		Horizontal vibration	3.86 (1.23)	2.38 (0.71)		2.50 (0.67)	< 0.001^b,c^	–	–
		Vertical vibration	4.00 (0.80)	2.52 (0.71)		2.41 (0.65)	< 0.001^b^	–	–
	Medial/lateral	–					0.522^a^	–	–
		Horizontal vibration	3.83 (1.26)	2.23 (0.79)		2.28 (0.71)	< 0.001^b^	–	–
		Vertical vibration	3.89 (1.79)	2.25 (0.79)		2.19 (0.71)	< 0.001^b^	–	–
Sajadi et al. [25]	0°	–					0.000^a,g^	–	1.4^g^
		LF	−2.91 (0.57)		−1.47 (0.88)		< 0.001^b^	–	1.4^f^
		HF	−2.51 (0.87)		−2.26 (0.99)		< 0.001^b^	–	−0.65^f^
	30°	–					0.6^a,g^	–	0.67^g^
		LF	13.6 (1.97)		14.6 (1.47)		0.05^b^	–	−0.9^f^
		HF	13.53 (1.67)		14.2 (1.54)		0.1^b^	–	−0.5^f^
	60°	–					0.06^a,g^	–	0.7^g^
		LF	29.7 (3.97)		28.48 (2.78)		0.04^b^	–	0.73^f^
		HF	29.2 (3.17)		28.63 (3.17)		0.2^b^	–	0.57^f^
Park et al. [26]	CoP velocity	–					0.039^a,h^	–	–
		SERW (*n* = 14)	4.69 (0.69)		3.50 (0.65)		0.000^b^	–	−1.19 (0.86)^f^
		SER (*n* = 14)	4.78 (0.71)		4.02 (0.79)		0.002^b^	–	−0.76 (0.75)^f^
		SE (*n* = 15)	4.81 (0.69)		4.35 (0.57)		0.010^b^	–	−0.46 (0.57)^f^
	CoP length	–					0.003^a,h^	–	–
		SERW (*n* = 14)	142.61 (2019)		108.41 (4.99)		0.000^b^	–	−34.20 (20.34)^f^
		SER (*n* = 14)	142.69 (2212)		128.83 (16.32)		0.027^b^	–	−15.29 (23.02)^f^
		SE (*n* = 15)	144.95 (22.37)		136.86 (16.13)		0.032^b^	–	−8.09 (12.57)^f^
	CoP area	–					0.048^a,h^	–	–
		SERW (*n* = 14)	9.79(2.33)		6.01(2.79)		0.000^b^	–	−3.78 (2.61)^f^
		SER (*n* = 14)	8.89 (2.60)		6.58 (2.65)		0.004^b^	–	−2.31 (2.45)^f^
		SE (*n* = 15)	9.29 (2.70)		7.87 (2.24)		0.037^b^	–	−1.42 (2.29)^f^

*STI* stability index, *SMI* sensorimotor Index, *SI* symmetry index, *PSTAntPost* postural stability in anterior–posterior, *PSTMedLat* postural stability in anterior–posterior, *CoP* center of pressure, *LF* low frequency, *HF* high frequency, *SERW* vibration + stabilization exercise + respiratory resistance, *SER* stabilization exercise + respiratory resistance, *SE* stabilization exercise

^a^between-group difference

^b^ingroup difference

^c^change by time

^d^treatment effect mean (95% CI)

^e^mean change from baseline

^f^pre versus post

^g^Greenhouse–Geisser correction

^h^F (p) Post hoc

#### Primary outcome: effect of WBVT on pain intensity

In the study by Pozo-Cruz et al. [18], a significant improvement in VAS scores was observed between the intervention group receiving WBVT and the control group receiving no therapy.

Similarly, Wang et al. [15] found a significant improvement in VAS scores between the intervention group receiving WBVT and the control group receiving general exercises. A significant improvement in the global perceived effect of change on the VAS was also analyzed between the groups [15].

Yang et al. [21] showed a significant improvement in VAS scores both between and within the groups. The intervention group received WBVT, while the control group received exercises for lumbar stability.

Karacay et al. [20] revealed a significant improvement in VAS scores both between and within the groups. The intervention group received WBVT, control group 1 received core stabilization exercises (CSG), and control group 2 (CG) received home exercises.

The study by Park et al. [26] also demonstrated a significant reduction in QVAS scores in all three groups (SERW, SER; SE). The SERW and SER groups showed a significant difference in QVAS improvement compared to the SE group.

Rittweger et al. [19] showed a significant reduction in VAS scores within both the intervention and control groups, but no significant difference was found between the intervention and control groups. The intervention group received WBVT, while the control group received lumbar extension exercises.

Micke et al. [23] observed a significant decrease in VAS scores in all groups (WBV, EMS, circuit training) before and after therapy, but there was no significant difference in pain reduction between the three groups.

Zheng et al. [22] observed a significant improvement during therapy in the single group using WBV.

Two studies compared different WBVT settings [24, 25]. In the study by Kim et al. [24], a significant improvement in VAS scores was found after both horizontal and vertical vibrations, but no significant difference was observed between the two groups.

The same result was observed in the crossover study by Sajadi et al. [25], where a significant improvement was seen within both low-frequency (LF) and high-frequency (HF) WBVT groups, but no significant difference was found between the groups.

#### Primary outcome: effect of WBVT on activities of daily living

A significant improvement in daily activity measured with the ODI was observed between the intervention group and control group in the studies by Kaeding et al. [17] and Pozo-Cruz et al. [18]. The intervention group received WBV, while the control group did not receive any treatment. Similarly, Wang et al. [15] reported a significant improvement in ODI scores in the intervention group compared to the control group, which received general exercises. A significant improvement in the global perceived effect of change on the ODI was also found between the groups.

However, Yang et al. [21] did not observe a significant improvement in ODI scores between the groups. Both the control group, which performed exercises for lumbar stability, and the intervention group with WBVT showed a significant improvement in the follow-up assessment.

Furthermore, a significant improvement was observed after WBVT with horizontal and vertical vibration in the study by Kim et al. [24]. However, no significant difference was found between the two groups.

In the study by Wegener et al. [16], no significant improvement in activities of daily living was observed in either the intervention group with WBVT or the control group with exercises using TheraBand® or weights.

#### Primary outcome: effect of WBVT on physical function

A significant improvement in physical function, as measured by the RMQ, was observed between the intervention and control groups in the studies by Pozo-Cruz et al. [18] and Kaeding et al. [17], where WBVT was compared to no therapy.

Similarly, in the comparison among the three groups in the study by Park et al. [26], both SERW and SER demonstrated a significant increase, surpassing the SE group.

In the study by Karacay et al. [20], significant improvement was observed in the groups receiving WBVT and core stabilization exercises with the therapists establishing and controlling the neutral position of the pelvis, but not in the group performing only home exercises. A significant improvement in RMQ scores between the groups was not determined.

#### Secondary outcome: effect of WBV on trunk proprioception

In the study by Wang et al. [15], the intervention group exhibited additional positive outcomes in terms of lumbar flexion joint position sense and lumbar extension joint position sense compared to the control group. Both groups received a general physical training program, with WBVT (intervention group) and without WBVT (control group).

Similarly, in the study by Zheng et al. [22], a significant reduction in lumbar flexion and extension angle deviation was observed following WBV.

In the study by Sajadi et al. [25], a significant improvement in reposition error (RE) was found in the low-frequency (LF) group at 0° lumbar flexion, 30° lumbar flexion, and 60° lumbar flexion, while in the high-frequency (HF) group, a significant improvement was observed only at 0° lumbar flexion. A significant difference between the two groups was observed only at 0° [25]. Based on the findings, it was observed that the variation in lumbar flexion RE was influenced by the frequency of WBVT rather than the angle of flexion [25]. The low-frequency WBV, as implemented in the proposed protocol, resulted in greater improvement in RE [25].

#### Secondary outcome: effect of WBVT on postural stability

Wegener et al. [16] examined postural stability using the MFT-S3 Check in patients with no back pain and NCBP before the intervention. Only patients with NCBP received an intervention, after which the MFT S3 Check was repeated. There was no difference in postural stability between the no back pain and NCBP groups in sitting or in standing positions before the intervention. After therapy, the control group, which performed trunk stability exercises using TheraBand® and weights, showed a significant improvement in the stability index (STI) in both standing and sitting positions, as well as in the sensorimotor index (SMI) in the standing position. The WBVT group did not show a significant improvement in postural stability, and there was also no significant difference between the groups.

The assessment of postural control in the study of Kaeding et al. [17], comparing participants in the intervention group receiving WBVT and participants without therapy, showed no significant difference between the groups before the WBVT therapy and even 3 months after its completion.

The fall index (FI) in the study by Yang et al. [21] showed a significant improvement in the vibration group compared to the group performing only lumbar stability exercises.

In the study by Pozo-Cruz et al. [18], a significant improvement in postural stability was observed in the intervention group receiving WBVT compared to the control group without therapy, as measured by the PSTAntPost.

Both groups in the crossover study by Kim et al. [24] showed a significant improvement in standing balance control scores with both horizontal and vertical vibration. However, there was no significant difference between the groups.

In the study by Park et al. [26] a significant improvement in balance ability was observed in the SERW group, while the SER and SE groups did not show a significant improvement.

## Discussion

Whole-body vibration therapy is a measure which is frequently used in the clinical setting. It seems to have several advantages for both patients as well as physicians. For example, it offers effective exercises that require minimal space and do not necessitate additional equipment. Additionally, the incidence of adverse events associated with WBVT therapy is rare, as none of the 12 examined studies reported any such occurrences. Moreover, this approach is not only beneficial for individuals who are unable to participate in traditional exercise therapy due to musculoskeletal constraints, but also for those who experience fears or anxieties related to conventional exercise therapy.

The term “kinesiophobia” is frequently encountered in the literature when discussing patients suffering from CLBP [27]. These patients avoid physical activity due to fear of experiencing painful movements or reinjury. They assume a position of avoidance and endure their pain through physical inactivity [24, 28].

However, literature has demonstrated that regular physical activity is superior to no treatment or usual care in managing CLBP [29].

Patients with CLBP experience significant limitations in their daily functioning and physical activity due to the persistent and ongoing pain, which affects their abilities in both professional and personal life [30]. An improvement in these limitations during the course of therapy may be attributed to a mere reduction in pain [19]. Effective pain reduction is the crucial initial step in the treatment of CLBP patients, leading to subsequent positive outcomes.

The findings from the present systematic review reveal an improvement in the pain condition among patients who underwent WBVT [15, 18–26]. Five out of the 10 studies demonstrated a significant superiority of the WBVT compared to the control group. Additionally, five studies showed a significant improvement in pain symptoms within the intervention group during the observation period.

The improvement in pain status and positive response to physical activity resulting from WBVT can lead to a reduction in kinesiophobia [27]. This is reflected in the results of our systematic review. Three out of the six studies demonstrated a significant improvement in pain symptoms between the intervention and the control group [15, 17, 18]. Two of the six studies showed a significant improvement within the intervention group during the observation period [20, 23].

Likewise, there was an improvement in physical function after WBVT. One study showed a significant improvement within the intervention group [20]. A significant improvement between the groups was detected in three of the four studies that examined physical function [17, 18, 20, 26].

The pathology of CLBP is difficult to understand due to its complex heterogeneity. Peripheral causes of CLBP can involve degeneration of the bony and ligamentous, fibrous, and muscular components of the lumbar spine. Nociceptors contained within these components can be sensitized through inflammatory processes, lowering their threshold for activation. Additionally, these processes can trigger pathological nerve growth, resulting in neuropathic pain [31]. However, central mechanisms are also described. This involves an alteration in the processing of stimuli in the brain, particularly with faulty descending pain pathways [31]. Structural differences in the musculature have also been identified in CLBP. Atrophy of the multifidus muscles and an abnormality in the fiber type of the paraspinal muscles have been reported in CLBP [32].

It is hypothesized that CLBP patients exhibit divergences in trunk muscle activity and kinematics, and the spinal stability may either be increased or decreased in this patient population [33]. A “tight control” can lead to high spinal loading with sustained spinal activity, while “loose control” can result in increased tensile loading [33]. Tailored treatments should be directed at these phenotypes based on the condition of spinal stability [33]. Our review investigates the impact of WBVT on proprioception and postural stability in patients with subacute and CLBP, taking into account the altered spinal kinematics observed in these individuals.

Two of the two studies that examined proprioception showed significant improvement in proprioception in patients with CLBP [15, 22].

It was observed that a low frequency of 30 Hz resulted in a more significant improvement in proprioception compared to a high frequency of 50 Hz [25].

The results regarding the effects of WBVT on postural stability in patients with subacute and CLBP are inconclusive in this systematic review. In three studies WBVT showed significant improvements compared to the control group [15, 18, 23], and one study showed significant improvements within the intervention group [21]. Nevertheless, there are two references that showed no significant improvement after WBVT [16, 17].

Overall, the results support the efficacy of WBVT in patients with subacute and CLBP with a particular focus on pain reduction and improvement in physical and daily activity. The contradictory results regarding postural control/stability reinforce the hypothesis that patients with CBP may exhibit either “tight control” or “loose control” of the spine, requiring specific therapies based on their condition. Further studies should classify patients into different groups based on the underlying etiology to determine significant differences in the effectiveness of WBV.

Of the 12 studies, one specifically focused on patients with subacute NLBP [26]. The results of these patients were found to be comparable to those of patients with CLBP. These findings suggest that WBVT may be beneficial even in the subacute phase of low back pain. Consequently, it can be inferred that WBVT has the potential to be applied early in the management of subacute NLBP.

Interestingly, many publications in the literature address work-related WBVT in relation to LBP. LBP particularly occurs during seated activities such as driving in vehicles [34–36].

A systematic review of Burström et al. [37] establishes a link between occupational vibration and LBP, identifying a spinal resonance frequency for the seated operator between 4 and 8 Hz.

However, the reason why WBVT is still offered as a treatment for LBP despite this association is a crucial difference in the duration and the therapeutic framework of the applied vibration [7].

In this systematic review, evidence suggests that WBVT represents a promising approach with potential benefits for the management of subacute and CLBP. It is essential to acknowledge the multifactorial nature of back pain etiology, thus requiring a multimodal treatment approach. Certain psychological factors such as fear-avoidance beliefs, self-efficacy, pain-coping mechanisms, catastrophizing tendencies, and even the presence of depressed mood have been shown to be important predictors of a person’s disability status [38]. Consequently, WBVT should be considered as a complementary therapy in clinical practice. Additionally, it is crucial to incorporate psychoeducation alongside therapeutic interventions.

There are several limitations to this systematic review. The comparability of the studies is restricted due to variations in study protocols, such as observation periods, frequency, and administration of WBVT. In future studies, it will be necessary to improve the methodological quality through minimizing the methodological limitations (for example, missing control group, low sampling rate, or no standardized clinical outcome). Furthermore, more high-quality studies are urgently needed to find the best WBVT for subacute and CLBP.

## Conclusion

Therapeutic whole-body vibration appears to be a viable and secure treatment modality for individuals experiencing subacute and CLBP. The use of WBVT as an adjunctive component within a multimodal treatment framework for subacute and CLBP, with an emphasis on pain reduction and enhancement of activities of daily living and physical functions, as well as improvements in postural stability and proprioception, has demonstrated beneficial effects. However, further investigation is necessary, with standardized assessments and interventions to explore optimal protocols, long-term effects, and the potential mechanisms underlying the observed positive outcomes associated with WBVT.
